# Vaccination coverage and timeliness among children in Ethiopia

**DOI:** 10.1136/bmjgh-2025-021447

**Published:** 2026-06-19

**Authors:** Clara Pons-Duran, Bezawit Mesfin Hunegnaw, Chalachew Bekele, Kassahun Alemu, Yayha Mohammed, Kathleen M Kurowski, Fiseha Tadesse, Melkamu Ayalew, Abraham Alebie, Lisanu Taddesse, Delayehu Bekele, Grace J Chan

**Affiliations:** 1 Department of Epidemiology, Harvard T H Chan School of Public Health, Boston, Massachusetts, USA; 2 HaSET Maternal and Child Health Research Program, Addis Ababa, Ethiopia; 3 Department of Pediatrics and Child Health, St Paul’s Hospital Millennium Medical College, Addis Ababa, Ethiopia; 4 Clinical Futures, The Children’s Hospital of Philadelphia, Philadelphia, Pennsylvania, USA; 5 Department of Obstetrics and Gynecology, Debre Birhan Referral Hospital, Debre Birhan, Ethiopia; 6 National Immunization Program, Federal Ministry of Health, Addis Ababa, Ethiopia; 7 Birhan HDSS, St Paul’s Hospital Millennium Medical College, Addis Ababa, Ethiopia; 8 Department of Obstetrics and Gynecology, St Paul’s Hospital Millennium Medical College, Addis Ababa, Ethiopia; 9 Department of Pediatrics and Epidemiology, University of Pennsylvania Perelman School of Medicine, Philadelphia, Pennsylvania, USA

**Keywords:** Epidemiology, Vaccines, Child health

## Abstract

Vaccinations are crucial for preventing and controlling infectious diseases. However, in Ethiopia, a significant proportion of children remain unimmunised. This study aimed to describe vaccination coverage among children in a field site in Ethiopia, and the proportion of children who were vaccinated on time according to the National Vaccination Schedule. We analysed data from a longitudinal study of 7417 children conducted in Ethiopia, which includes a health and demographic surveillance system (HDSS) with house-to-house surveillance visits every 3 months. The study population was children born between 2018 and 2021 enrolled in the HDSS. Vaccination data were collected through standardised questionnaires, which abstracted information from vaccination cards and reports from caregivers. We used two analytical approaches to calculate the vaccination coverage of the full package of recommended vaccines, the coverage of each specific recommended vaccine and timeliness of vaccine administration. Using a longitudinal approach, 26% (2018) to 31% (2021) of children were fully vaccinated. Using a cross-sectional survey approach, 32% (2018) to 42% (2021) were fully vaccinated. Similarly, coverage of specific vaccines is lower in the longitudinal approach compared with cross-sectional surveys. Using the longitudinal and cross-sectional strategies, 76% and 67% of children, respectively, who received the measles vaccine before 12 months of age were vaccinated within 4 weeks of becoming eligible. Additionally, about 40% of children received the third doses of oral poliovirus, pentavalent and pneumococcal vaccines, as well as the second dose of rotavirus vaccine, within 4 weeks of eligibility. In this study, vaccination coverage in the study area was low, with fewer than half of infants receiving their vaccinations within 4 weeks of the recommended administration time. Estimates of vaccination coverage with longitudinal data compared with cross-sectional data provide a more accurate assessment of vaccine coverage and timeliness and support more precise monitoring of immunisation efforts.

**WHAT IS ALREADY KNOWN ON THIS TOPIC**

- Vaccination coverage is a high priority for the Ministry of Health in Ethiopia. Current estimates of coverage levels are suboptimal. The timeliness of vaccine administration and precise documentation of vaccination delays have not been well studied, particularly using longitudinal data.

**WHAT THIS STUDY ADDS**

- Vaccination coverage levels are likely even lower than currently estimated due to recall and survivor biases from cross-sectional surveys. This study provides the first direct comparison of cross-sectional survey and birth cohort approaches for measuring vaccine coverage and timeliness in a rural Ethiopian setting. Our findings show that survey-based analyses systematically overestimate both coverage and timeliness compared with longitudinal cohort analyses, and that significant delays persist for most vaccines, particularly BCG.

**HOW THIS STUDY MIGHT AFFECT RESEARCH, PRACTICE OR POLICY**

- These results highlight the need to incorporate longitudinal surveillance or electronic registries into national immunisation monitoring to obtain more accurate estimates of vaccine coverage. Adopting such approaches, along with standardised definitions for coverage and timeliness, can inform more targeted and effective vaccination strategies and policymaking, especially in low-resource settings.

## Introduction

 Vaccine-preventable diseases are leading causes of child mortality, especially in sub-Saharan Africa.^1^ In Ethiopia, pneumonia, lower respiratory tract infections, diarrhoea and measles are major contributors to under-5 mortality.^2^ Vaccinations are essential for preventing and controlling infectious diseases, reducing epidemic risk and saving millions of lives annually.^3^ Ethiopia has continued to make progress in childhood immunisation by establishing the Ethiopian Immunisation Technical Advisory Group and implementing the Expanded Programme on Immunisation (EPI), increasing funding and vaccine availability. However, many Ethiopian children remain underimmunised, reflecting a broader global challenge. In 2019, only 40% of children aged 12–23 months received the WHO or Ministry of Health (MoH) or recommended set of vaccines by their first birthday: one dose of BCG, three doses of DPT-HepB-Hib (pentavalent), three doses of oral polio vaccine (OPV) and one dose of measles-containing vaccine.^4^

Vaccination coverage is commonly measured in two ways.^5^ The first approach tracks whether children receive vaccines, irrespective of the timing. The second approach focuses on timeliness and measures whether vaccines are administered according to the national schedules. Timeliness is particularly relevant for assessing actual protection offered by vaccines during infancy, since most coverage data record only immunisation status at 12 months of age rather than when individual vaccines were received.^5^ There is limited information regarding the timing of specific vaccinations and about delays for each recommended vaccine.

In Ethiopia and similar low-resource settings, coverage is usually assessed through cross-sectional surveys of children 12–23 months old.^6^ Such surveys may overestimate protection due to survivor and recall biases.^5^ With no systematic vaccine registry to facilitate precise and continuous tracking of immunisation status, and without studies comparing results between longitudinal and cross-sectional survey approaches, significant knowledge gaps persist in vaccination coverage and timeliness trends. Addressing these gaps would support operational strategies within the Ethiopian National Immunisation Technical Advisory Group and potentially improve the National Implementation Guideline of the EPI.^7^ Persistent discrepancies in coverage estimates underscore the need to improve current measurement methods.^8^

This study leverages a well-established site with longitudinal data to assess full vaccination rate and coverage of specific vaccines among children in Ethiopia from 2018 to 2021 and to quantify the proportion vaccinated on time per the National Vaccination Schedule. The longitudinal design enables a comparison of two approaches: a cross-sectional survey and a birth cohort analysis. The birth cohort analysis effectively simulates a vaccination registry, providing a better understanding of coverage and timeliness.

## Methods

### Study design and setting

To address these aims, we used data from the Birhan field site in Amhara, Ethiopia, composed of 16 kebeles with 77 766 individuals.^9^ The site features a health and demographic surveillance system (HDSS) with house-to-house visits every 3 months. Established in 2018, it supports community and facility-based research on maternal and child health across rural highland and lowland areas in two districts (online supplemental figure S1).^9^

Vaccination data were collected during surveillance rounds using child health questionnaires administered by trained data collectors at the homes of participants. Data collectors abstracted data from a vaccination card, and if not available, asked mothers and caregivers about vaccination history.^9^ In these cases, vaccination dates were not collected. Questions on vaccination were adapted from the Ethiopia Demographic Health Survey.^4^

### Study population

The study included 7417 children born between 2018 and 2021 and enrolled in the Birhan HDSS before age 24 months (September 2018–April 2023). Of the 55 months of surveillance, we analysed data from 33 months of complete surveillance; the remaining months had incomplete data due to COVID-19 and local conflict events.

### Study outcomes and definitions

According to the Ethiopian MoH’s Health Management and Information System indicators reference guide,^10^ a child was considered fully vaccinated after receiving one dose of BCG, three doses of OPV, three doses of DPT-HepB-Hib, three doses of pneumococcal conjugate vaccine (PCV), two doses of rotavirus vaccine, one dose of measles vaccine and one dose of inactivated polio vaccine (IPV) by their first birthday. Online supplemental table S1 summarises the current EPI vaccination schedule in Ethiopia for the first year of life and lists the WHO-recommended vaccines not yet included in the Ethiopian EPI as of March 2024.

We report vaccination coverage and timeliness via a range of metrics. Primary metrics include: (1) full vaccination coverage, defined as the proportion of children who received all vaccines required for full immunisation in Ethiopia (BCG, OPV, DPT-HepB-Hib, PCV, rotavirus, measles); (2) coverage of specific vaccines, quantified as the proportion of children who completed the full regimen of each vaccine and (3) timeliness, defined as the proportion of children who received all doses of a specific vaccine within a 4-week recommended schedule. The 4-week window accounts for possible delays due to environmental and logistics challenges. IPV data were not collected as it was not routinely included in Ethiopia’s EPI programme evaluations and was generally used to monitor specific polio immunisation programmes.

Additional secondary metrics include: (1) the proportion of zero-dose children, defined as the percentage of children who did not receive the first dose of pentavalent vaccine by the end of their first year of life, following Gavi’s operational definition^11^ and (2) the drop-out rate, measured as the proportion of children who received the first dose of pentavalent vaccine but did not subsequently receive either the third dose of pentavalent vaccine or the first dose of measles vaccine, among those with available follow-up data in the HDSS.

Reporting of this study has been conducted according to the Strengthening the Reporting of Observational Studies in Epidemiology (STROBE) guidelines presented in online supplemental table S2.

### Statistical analysis

Since most study participants were observed in multiple HDSS rounds, we used the same study population and applied two existing, rigorous methodological approaches, adapted from Adetifa *et al*.^5^ An overview of these approaches for each primary and secondary outcome is provided in online supplemental tables S3 and S4.

The first approach, the ‘birth cohort approach’, is a proxy for a continuous electronic vaccination registry. The denominator was the number of children who survived to the age of vaccine eligibility (for specific vaccines) or 12 months (for full vaccination coverage), by year of birth cohort. The numerator was the number of vaccinated before 12 months of age, for cohorts from 2018 to 2021.

The second, the ‘survey approach’, mimics a cross-sectional survey, with vaccination status assessed on 1 January each year from 2020 to 2023.^5^ The denominator includes children aged 12–23 months on that date in each year. The numerator was the number of children with the required 0–12-month vaccinations before the data collector-administered interview and before 24 months of age. Data for this approach are from multiple rounds of data collection for children aged 12 to 23 months old. Online supplemental figure S2 summarises the study design and estimation links for both approaches.

Both approaches were used to estimate timeliness of full-vaccine administration. Timeliness analysis required both the child’s date of birth and vaccination administration date, sourced from vaccination cards, and analysis was restricted to children with this information. A dose was considered timely if administered within 28 days of the recommended age per the Ethiopian immunisation schedule proposed by Luman *et al* and the WHO guidelines.^12 13^ We compared characteristics of children with and without vaccination cards (online supplemental table S5), noting differences in socioeconomic status and proximity to health facilities, which may affect generalisability. For timeliness, denominators included vaccinated children, and numerators were those vaccinated within 4 weeks of vaccine eligibility. Only children with available vaccination cards and dates were included. To assess selection bias, we performed a descriptive analysis comparing children with and without vaccination cards on socioeconomic and demographic variables, such as proximity to the nearest health facility, wealth index, type of kebele and woreda.

Zero-dose and drop-out proportions were estimated using both approaches. For zero-dose estimates, denominators included children eligible to receive the first pentavalent dose, and numerators were those not vaccinated before 12 months (birth cohort) or before interview (survey). For dropouts, denominators included children who received a first pentavalent dose; numerators were those missing the third pentavalent or measles vaccine.

For all outcomes, descriptive statistics were calculated, including frequencies, proportions and Agresti-Coull 95% CIs.^14^ Analyses were conducted using Stata V.17.^15^

## Results

Using both the ‘birth cohort’ approach and ‘survey’ approach, we analysed data from 7417 children born and visited in at least one round of the Birhan HDSS between September 2018 and April 2023, which included 33 months of complete surveillance and compromised all eligible live births identified within the HDSS database across 111 rounds of data collection. Of these, 35 children (<1%) had missing data on complete receipt of vaccination and were not included in the analysis, and 4357 (59%) had a vaccination card at the time of interview, though card availability completeness varied across vaccines and HDSS rounds. Over 90% of children with cards had all vaccination dates recorded. Table 1 presents their sociodemographic characteristics.

**Table 1:** Characteristics of children of the Birhan field site born between 2018 and 2021 (N=7417)

Characteristics		n (%)
District (woreda)	Angolela	2827 (38)
	Kewet	4590 (62)
Type of residence	Urban	1461 (20)
	Rural	5956 (80)
Source of income	Farming	5800 (78)
	Other sources	1394 (19)
	Missing	223 (3)
Nearest health facility (HF)	Chacha Health Centre	1752 (24)
	Tsigereda Health Centre	997 (13)
	Abaya atir Health Centre	2055 (28)
	Tere Health Centre	1187 (16)
	Shewarobit Health Centre	336 (5)
	Shewarobit Hospital	819 (11)
	Other	59 (1)
	Missing	212 (2)
Walking time to nearest HF	<30 min	1660 (22)
	≥30 min	5545 (75)
	Missing	212 (3)

Online supplemental table S6 contains the specific sample sizes for each year and outcome calculation using the birth cohort and survey approaches. Among the 7382 live births from 2018 to 2021, 6525 children (88%) were included in the survey approach and 2495 children (34%) were included in the birth cohort approach. This proportion of the birth cohort varied by birth year, from 13% in 2019 to over 50% in 2021, reflecting disruptions in follow-up outside of our control, such as the COVID-19 pandemic.

**Figure 1:**
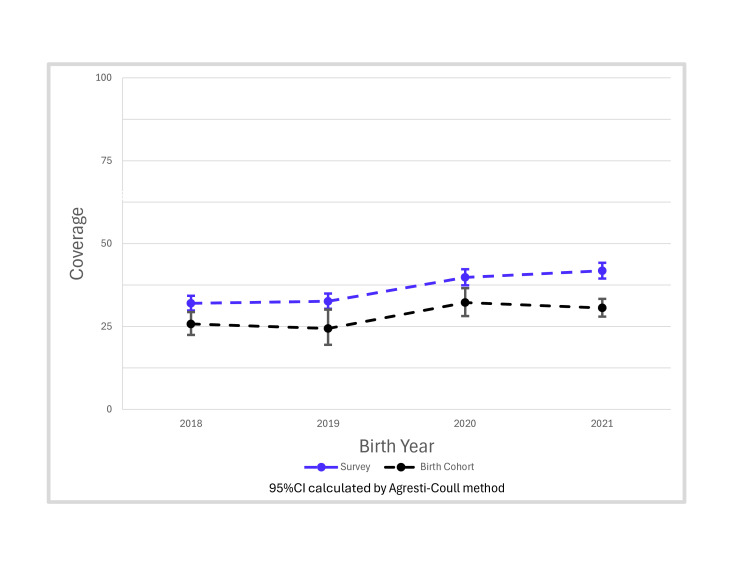
Full-vaccination coverage by birth cohort and survey population aged 12–23 months over time.

From figure 1 and table 2, the proportion of fully immunised children ranged from 26% (2018) to 31% (2021) using the birth cohort approach, and from 32% (2018) to 42% (2021) using the survey approach. Coverage rates for individual vaccines also increased across birth year cohorts. By 2021, coverage exceeded 50% for all vaccines except measles, which reached 39% based on the birth cohort approach. Survey-based estimates indicated even higher vaccine coverage, with most vaccine rates nearing 60% and BCG reaching 71%. Survey results were consistently higher than cohort-based estimates; for example, in 2021, measles vaccine coverage was 55% in the survey compared with 39% by birth cohort approach—a 16% difference. Full immunisation coverage in 2021 was 42% according to survey results, compared with 31% by the birth cohort approach, an 11% difference.

**Table 2:** Vaccination coverage among children of the Birhan field site by birth cohort and survey population aged 12–23 months, over time

Vaccine	Birth cohort	N	Coverage birth cohort (%, 95% CI)	Survey date	N	Coverage survey 12–23 months (%, 95% CI)
BCG	2018	619	43 (39 to 47)	1 January 2020	1694	47 (44 to 49)
	2019	250	38 (32 to 44)	1 January 2021	1616	44 (41 to 46)
	2020	477	48 (44 to 53)	1 January 2022	1567	55 (52 to 57)
	2021	1148	62 (59 to 64)	1 January 2023	1648	71 (68 to 73)
OPV (3 doses)	2018	617	36 (33 to 40)	1 January 2020	1694	39 (36 to 41)
	2019	250	35 (30 to 41)	1 January 2021	1616	39 (36 to 41)
	2020	475	43 (38 to 47)	1 January 2022	1567	47 (45 to 49)
	2021	1148	51 (48 to 53)	1 January 2023	1648	58 (56 to 61)
Pentavalent (3 doses)	2018	617	35 (32 to 39)	1 January 2020	1694	39 (37 to 41)
	2019	250	34 (28 to 40)	1 January 2021	1616	38 (36 to 41)
	2020	475	42 (38 to 47)	1 January 2022	1567	48 (45 to 50)
	2021	1148	52 (49 to 55)	1 January 2023	1648	61 (59 to 63)
PCV (3 doses)	2018	617	36 (32 to 40)	1 January 2020	1694	39 (36 to 41)
	2019	250	34 (28 to 40)	1 January 2021	1616	38 (35 to 40)
	2020	475	42 (38 to 47)	1 January 2022	1567	48 (45 to 50)
	2021	1148	52 (49 to 55)	1 January 2023	1648	61 (58 to 63)
Rotavirus (2 doses)	2018	617	39 (35 to 43)	1 January 2020	1694	40 (38 to 42)
	2019	250	34 (28 to 40)	1 January 2021	1616	39 (36 to 41)
	2020	475	45 (40 to 49)	1 January 2022	1567	51 (48 to 53)
	2021	1148	57 (54 to 60)	1 January 2023	1648	65 (63 to 67)
Measles	2018	617	33 (29 to 37)	1 January 2020	1694	38 (36 to 41)
	2019	250	30 (25 to 36)	1 January 2021	1616	38 (36 to 41)
	2020	475	35 (31 to 40)	1 January 2022	1567	46 (44 to 49)
	2021	1148	39 (36 to 42)	1 January 2023	1648	55 (52 to 57)
Full vaccination	2018	617	26 (23 to 29)	1 January 2020	1694	32 (30 to 34)
	2019	250	24 (20 to 30)	1 January 2021	1616	33 (30 to 35)
	2020	475	32 (28 to 37)	1 January 2022	1567	40 (37 to 42)
	2021	1147	31 (28 to 33)	1 January 2023	1648	42 (40 to 44)

OPV, oral polio vaccine; PCV, pneumococcal conjugate vaccine.

Delays in vaccination according to schedule were common among children with a vaccination card with documented dates. The measles vaccine was most often administered on time, although there were more delays using the survey approach. Three-quarters of measles-vaccinated children in the birth cohort approach received it within 4 weeks of eligibility, while 67% did so in the survey approach (table 3). For other vaccines (OPV, pentavalent, PCV, rotavirus), only 40% received the final doses within 4 weeks of vaccine eligibility. Both approaches produced similar timing results. BCG experienced the greatest delays, with only 5%–6% receiving it within 4 weeks of birth, although timeliness improved in 2020. Children with vaccination cards were more often from the wealthier households, living in rural areas of Angolela woreda, and within 30 min of a health facility (online supplemental table S5).

**Table 3:** Timely vaccination coverage among children of the Birhan field site by birth cohort and survey population aged 12–23 months, over time

Vaccine	Birth cohort	N	Timely vaccination (%, 95% CI)*	Survey date	N	Timely vaccination (%, 95% CI)*
BCG	2018	198	5 (2 to 9)	1 January 2020	590	5 (4 to 8)
	2019	81	5 (2 to 12)	1 January 2021	501	6 (4 to 8)
	2020	178	6 (3 to 11)	1 January 2022	631	5 (3 to 7)
	2021	590	6 (5 to 9)	1 January 2023	990	6 (5 to 8)
OPV (three doses)	2018	195	42 (35 to 49)	1 January 2020	571	43 (39 to 47)
	2019	82	43 (33 to 54)	1 January 2021	504	44 (40 to 48)
	2020	167	45 (38 to 53)	1 January 2022	583	42 (38 to 46)
	2021	506	39 (35 to 43)	1 January 2023	861	39 (36 to 43)
Pentavalent (three doses)	2018	194	42 (35 to 49)	1 January 2020	577	43 (39 to 47)
	2019	80	40 (30 to 51)	1 January 2021	517	44 (40 to 48)
	2020	164	42 (34 to 49)	1 January 2022	601	40 (37 to 44)
	2021	536	38 (34 to 42)	1 January 2023	917	39 (36 to 42)
PCV (three doses)	2018	192	40 (33 to 47)	1 January 2020	561	42 (38 to 46)
	2019	79	39 (29 to 50)	1 January 2021	453	44 (40 to 49)
	2020	95	50 (40 to 59)	1 January 2022	457	42 (37 to 46)
	2021	533	38 (34 to 42)	1 January 2023	910	39 (36 to 42)
Rotavirus (two doses)	2018	120	43 (34 to 52)	1 January 2020	501	47 (43 to 51)
	2019	78	41 (31 to 52)	1 January 2021	469	44 (40 to 49)
	2020	113	50 (41 to 60)	1 January 2022	510	47 (43 to 52)
	2021	595	39 (35 to 43)	1 January 2023	990	41 (38 to 44)
Measles	2018	158	77 (69 to 83)	1 January 2020	496	68 (63 to 72)
	2019	59	75 (62 to 84)	1 January 2021	403	72 (67 to 76)
	2020	78	81 (71 to 88)	1 January 2022	413	65 (60 to 70)
	2021	351	76 (71 to 80)	1 January 2023	710	67 (63 to 70)
Full vaccination	2018	–	–	1 January 2020	–	–
	2019	–	–	1 January 2021	–	–
	2020	–	–	1 January 2022	–	–
	2021	–	–	1 January 2023	–	–

* Timely vaccination: proportion of vaccinated children with available vaccination card and recorded vaccination dates who received their vaccines within 4 weeks of becoming age-eligible for vaccination.

-, NA (Not applicable); OPV, oral polio vaccine; PCV, pneumococcal conjugate vaccine.

Zero-dose rates were high (50%–60%) in 2018–2020, dropping to 36% (birth cohort) and 29% (survey) in 2021. Drop-out rates (pentavalent first dose to measles) decreased from 2018 to 2020 but increased in 2021 to 39% (birth cohort) and 28% (survey) (table 4).

**Table 4:** Rates of zero-dose children and vaccination drop-out rates among children of the Birhan field site by birth cohort and survey population aged 12–23 months, over time

Vaccine	Birth cohort	N	Birth cohort rate (%, 95% CI)	Survey date	N	Survey 12–23 months rate (%, 95% CI)
Zero-dose	2018	619	56 (52 to 60)	1 January 2020	1694	54 (52 to 56)
	2019	249	60 (54 to 66)	1 January 2021	1601	56 (54 to 59)
	2020	465	50 (46 to 55)	1 January 2022	1539	47 (44 to 49)
	2021	1106	36 (34 to 39)	1 January 2023	1622	29 (27 to 31)
Drop-out: pentavalent 1 to pentavalent 3	2018	273	21 (16 to 26)	1 January 2020	779	15 (13 to 18)
	2019	99	14 (9 to 23)	1 January 2021	698	12 (9 to 14)
	2020	231	13 (10 to 19)	1 January 2022	820	9 (7 to 11)
	2021	705	15 (13 to 18)	1 January 2023	1151	13 (11 to 15)
Drop-out: pentavalent 1 to measles	2018	273	26 (22 to 32)	1 January 2020	779	18 (15 to 20)
	2019	99	23 (16 to 33)	1 January 2021	698	13 (11 to 15)
	2020	231	28 (22 to 34)	1 January 2022	820	16 (14 to 19)
	2021	705	39 (36 to 43)	1 January 2023	1149	28 (25 to 30)

## Discussion

Coverage of full vaccination among infants born between 2018 and 2021 was low in the Birhan field site, regardless of the methodological approach. While coverage for full-dose regimens of individual vaccines was higher, rates remained below levels necessary for comprehensive protection against epidemic-prone diseases in infants. Prior studies in Ethiopia and the Amhara region have reported somewhat higher coverage rates than those observed in our analysis. A 2023 mixed-methods study in Amhara reported 56% full vaccination coverage using facility registers and tally sheets.^16^ Earlier cross-sectional surveys from 2016 and 2017 in Amhara reported higher coverage rates (58% to 77%).^17^,^19^ In contrast, the re-analysis of the 2019 Ethiopian Mini-Demographic and Health Survey data, using the most updated definition of full immunisation in the country, revealed a nationwide coverage of only 39%.^20^

Disparities between prior evidence and our findings are likely to reflect differences in study methodology and data sources. Previous studies mainly relied on cross-sectional surveys or complete facility records, which can introduce selection bias.^16^ In contrast, the Birhan field site is predominantly rural with many children residing far from health facilities, limiting their access to immunisations. During the study period, the COVID-19 pandemic and local conflicts disrupted service, which may have led to vaccine stock-outs and unstable distribution, resulting in further increased drop-out rates among children who started vaccination before a period of instability.^21^ Our findings reflect both the challenges of service delivery in remote areas and the broader impacts of instability.

Vaccine timeliness was suboptimal in Birhan. Except for the measles vaccine, fewer than half of vaccinated children received their vaccines within 4 weeks of the recommended age. BCG, which should be given at birth, was the most delayed with ~95% of children receiving it after 4 weeks. Quantification of the magnitude of immunisation delays is generally missing in Ethiopia.^6^ Most studies evaluating vaccine coverage employ cross-sectional designs which cannot assess timeliness of individual vaccines. Our findings regarding the widespread delay in receiving the BCG vaccine are novel in Ethiopia and could serve as valuable information for the MoH for the implementation and evaluation of future vaccination programmes and policies to achieve higher coverage and timeliness. Delays in BCG vaccination could be related to birth order; prevalence of zero-dose and under-immunised children increases as the number of siblings increases and firstborn children have a higher likelihood of full vaccination compared with children born after.^22^,^24^ In several studies on BCG vaccination, higher maternal education has been shown to positively impact childhood vaccination rates and increase coverage.^24^,^26^ In our study population, the majority of women reported primary education as their highest level of completed education. The delay in BCG vaccination we see could also be due to location of children; studies have shown that children who reside in more rural areas are more likely to have delayed BCG immunisations.^25 27^

Operational indicators such as zero-dose and drop-out rates are essential for monitoring immunisation coverage and adherence in low-resource settings. Our results show suboptimal zero-dose rates, similar to UNICEF’s 2021 estimate of 30%,^28^ although still higher than the 23%–24% observed in previous years.^29^ However, within our study period, we observed a decline in zero-dose rates during 2020 and 2021, suggesting a potential improvement at the local level. There were several programmes and initiatives implemented by the MoH during this period aimed at reducing the number of zero-dose children, including the implementation of the Periodic Intensification of Routine Immunisation campaign and Transform: Primary Health Care project. These programmes improved capacity building in the area and provided resources for training and data collection to improve reproductive, maternal, newborn and child health in Ethiopia.^30^,^32^ Dropout rates in Birhan followed patterns seen in other studies conducted in the Amhara region^17^ highlight the ongoing need for targeted interventions and robust follow-up.^18 19^

Using two different approaches—birth cohort and survey—provided valuable information on the use of cross-sectional surveys to monitor vaccine coverage. Survey results consistently overestimated coverage by 7% to 15% compared with birth cohort-based estimates. This finding aligns with the results from a similar comparison study in Kenya.^5^ This systematic overestimation of vaccination rates in our study may be explained by recall bias, as families may inaccurately recall the vaccine types and dosage, and by survivor bias since children who die before the survey are not captured. We recommend greater use of longitudinal data sources such as vaccine registries or cohort studies to complement cross-sectional surveys. While surveys remain valuable, integrating them with longitudinal approaches may improve accuracy and reduce biases in monitoring vaccination coverage, especially in low-resource settings. Using a longitudinal approach, such as vaccine registries or cohort studies, minimises survivor and recall biases by timely recording vaccination information at more frequent intervals, reducing the reliance on caregiver memory for vaccination status. Longer recall periods used in cross-sectional approaches could lead to incorrect or more missing vaccine doses, affecting vaccine coverage calculations. Using longitudinal data captures participants who may be lost in cross-sectional data, such as those that do not survive to be captured in a cross-sectional analysis, reducing survivor bias. Longitudinal datasets are more likely to obtain accurate measures of coverage and timeliness of vaccination in low-resource settings.

Our study highlights the need to expand efforts to improve vaccination coverage in Ethiopia. In the absence of adequate full immunisation rates, monitoring the administration of each recommended vaccine separately will inform policy and operational decisions. Furthermore, evidence is generally inconsistent across studies within Ethiopia.^8^ Therefore, we encourage the public health and academic community to standardise measurements and definitions concerning vaccine coverage to better compare across regions and evaluate progress over time using consistent indicators.

Some limitations should be considered when interpreting our results. Our coverage estimates, while lower than national and regional surveys, likely provide a more accurate picture of vaccination timing due to detailed cohort follow-up. However, only about one-third of eligible children were observed in the key age window due to circumstances outside of our control such as the COVID-19 pandemic, and these children tend to live closer to health facilities and possess vaccination cards, which may underestimate delays and those with no access to services. This highlights both the strengths of our longitudinal HDSS approach in capturing timely uptake and the challenges of ensuring cohort representativeness due to follow-up constraints. The Birhan field site offers valuable opportunities for community-level cohort analyses and may serve as a foundation for future studies that may implement an electronic vaccine registry in an LMIC setting such as Ethiopia. Practical challenges, such as missed visits and reliance on caregiver recall for undocumented vaccinations, are common in community-based studies and reflect real-world conditions in rural settings. IPV was not included in our definition of full vaccination coverage; although this does not affect comparisons between the birth cohort and survey approaches, which used the same definition, it may result in higher estimates relative to definitions that include IPV. Our findings mainly represent children with documented vaccination, likely underestimating actual delays among the most marginalised. Despite these challenges, the HDSS platform enables future analysis of individual and household-level factors, such as maternal education, socioeconomic status, and geographic access, to better identify at-risk populations and inform targeted interventions. Our approach demonstrates how longitudinal systems can provide actionable insights to strengthen immunisation programmes in resource-limited settings.

## Conclusions

In this cohort study, we found low vaccination coverage rates in the Birhan field site, where many infants did not receive the complete set of recommended vaccines under the Ethiopian EPI schedule. Fewer than half of infants received vaccinations within 4 weeks of the recommended timeframe. Our findings highlight the need for further research to understand the underlying causes of inadequate coverage, such as geographic barriers, limited health facility access or vaccine supply challenges. Incorporating longitudinal data collection alongside traditional cross-sectional surveys could enhance the precision of vaccine coverage and timeliness estimates, supporting more effective monitoring and programme planning at local and national levels.

## Supplementary material

10.1136/bmjgh-2025-021447 online supplemental figure 1

10.1136/bmjgh-2025-021447 online supplemental figure 2

10.1136/bmjgh-2025-021447 online supplemental file 1

## Data Availability

Data are available in a public, open data access repository.
