# Supplementary figures and images for: Vaccination coverage and timeliness among children in Ethiopia
Source: BMJ Glob Health. 2026 Jun 19;11(6):e021447. doi: 10.1136/bmjgh-2025-021447 (PMC13289158; doi:10.1136/bmjgh-2025-021447)

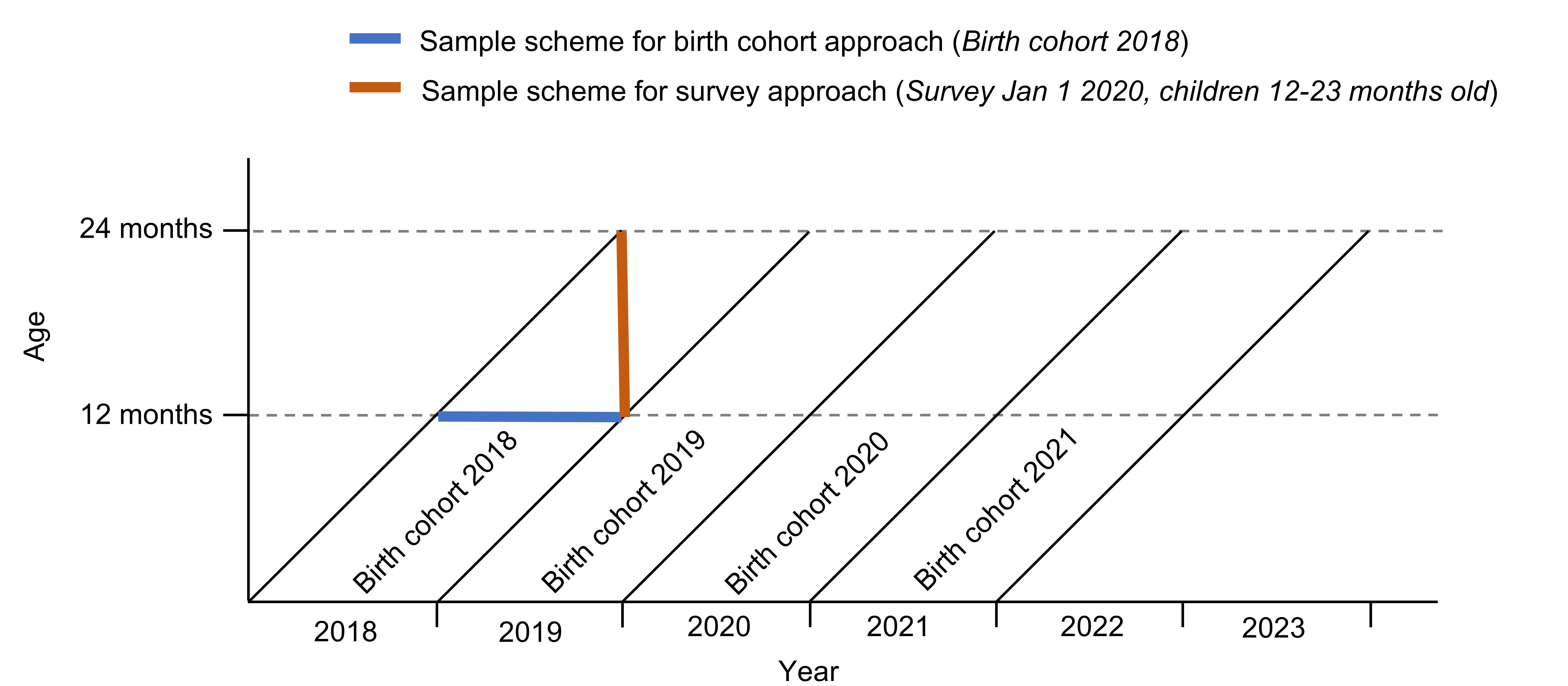

Supplement: online supplemental figure 2 [file bmjgh-11-6-s002.jpg]
