# Supplementary material for: Vaccination coverage and timeliness among children in Ethiopia
Source: BMJ Glob Health. 2026 Jun 19;11(6):e021447. doi: 10.1136/bmjgh-2025-021447 (PMC13289158; doi:10.1136/bmjgh-2025-021447)
Supplement: online supplemental file 1 [file bmjgh-11-6-s003.docx]

**Figure captions**

**Supplemental Figure S1**. Map of Birhan Health and Demographic Surveillance System study area [9].

**Supplemental Figure S2**. Summary of study design, sampling populations and estimation links for the birth cohort approach and the survey approach.

**Supplemental Table S1.** Schedule of vaccines included and not included in the Expanded Program on Immunization in Ethiopia for the first year of life as of March 2024.

| **Vaccine** | **Birth** | **6 weeks** | **10 weeks** | **14 weeks** | **6 months** | **9 months** |
| --- | --- | --- | --- | --- | --- | --- |
| Bacille Calmette-Guérin (BCG) | X |  |  |  |  |  |
| Bivalent oral polio vaccine (bOPV) | X | X | X | X |  |  |
| Inactivated polio vaccine (IPV) |  |  |  | X |  | X^±^ |
| Hepatitis B – birth dose (HepB-DB) | X*^‡^ |  |  |  |  |  |
| Pentavalent vaccine: diphtheria, tetanus and pertussis – Hepatitis B – *Haemophilus influezae* type b (DPT-HepB-Hib) |  | X | X | X |  |  |
| Pneumococcal conjugate vaccine (PCV) |  | X | X | X |  |  |
| Rotavirus vaccine |  | X | X |  |  |  |
| Measles vaccine |  |  |  |  |  | X |
| Rubella-containing vaccine (RCV) |  |  |  |  |  | X^†^ |
| Yellow fever vaccine |  |  |  |  |  | X^§‡^ |
| Typhoid vaccine (optimally typhoid conjugate vaccine, TCV) |  |  |  |  |  | X^¶‡^ |
| Meningococcal vaccine (optimally Meningitis A conjugate vaccine) |  |  |  |  |  | X^#‡^ |

Shaded cells: vaccines not included in the Expanded Program on Immunization of Ethiopia.

* Hepatitis B birth dose (HepB-BD) was recently recommended in 2021 [10], but not included in the Expanded Program of Immunization. It has been recently piloted, and the National Implementation Guideline of the EPI states that this vaccine will be introduced in the near future [7].

± The second dose of IPV is planned to be incorporated into the EPI in early-mid 2024.

† The WHO recommends that countries that have not yet introduced a rubella-containing vaccine (RCV) into their immunization programs should do so if they can achieve a coverage level of 80% or greater, through either routine immunization or campaigns [28].

§ A yellow fever risk assessment was conducted in Ethiopia and identified risk areas that require the introduction of the yellow fever vaccine in the future [29]. The National Implementation Guideline of the EPI states that this vaccine will be introduced in the near future [7].

¶ The WHO recommends typhoid conjugate vaccine (TCV) to be administered at the same time as other vaccine visits at 9 months of age or in the second year of life [28]. The National Implementation Guideline of the EPI states that this vaccine will be introduced in the near future [7].

# Guided by a risk assessment and to eliminate Meningitis A, Ethiopia conducted the catch-up Meningitis A supplemental immunization activities in 3 phases from 2013-2015 [29]. The National Implementation Guideline of the EPI states that this vaccine will be introduced in the near future [7].

‡ Planned for future introduction but were not yet part of the routine EPI schedule during the study period.

**Supplemental Table S2**. STROBE Checklist: Cross-sectional and Longitudinal Observational Study

| **Item No.** | **STROBE Checklist Item** | **Manuscript Section / Page Number** |
| --- | --- | --- |
| **Title and Abstract** |  |  |
| 1 | Indicate study design in title or abstract using a commonly used term | Abstract (Page 2) |
| 2 | Provide an informative and balanced abstract summarizing objectives, methods, results, and conclusions | Abstract (Page 2) |
| **Introduction** |  |  |
| 3 | Explain the scientific background and rationale for the study | Introduction (Pages 5-6) |
| 4 | State specific objectives and any prespecified hypotheses | Introduction – Final paragraph (Page 6) |
| **Methods** |  |  |
| 5 | Present key elements of the study design early in the paper | Methods – Overview (Page 6-7) |
| 6a | Describe setting, location, and dates, including relevant periods of recruitment and follow-up | Methods – Study setting (Page 6-7) |
| 6b | Describe eligibility criteria and methods of participant selection | Methods – Sampling and population (Page 7) |
| 7 | Clearly define all outcomes, exposures, predictors, and confounders | Methods – Outcomes and definitions (Page 7-8) |
| 8 | For each variable, give data sources and measurement methods | Methods – Data sources and measurement (Page 6-7) |
| 9 | Describe efforts to address potential sources of bias | Methods – Statistical analysis (Page 8-9); Supplementary File |
| 10 | Explain how study size was arrived at | Methods – Data sources/sampling (Page 6-7); also shown in Results (Page 9-10) |
| 11 | Explain how quantitative variables were handled in the analyses | Methods – Statistical analysis (Page 8-9) |
| 12a | Describe all statistical methods including those to control for confounding | Methods – Statistical analysis (Page 8-9); Supplementary File |
| 12b | Describe any methods used to examine subgroups and interactions | Not applicable/not included |
| 12c | Explain how missing data were addressed | Supplemental File |
| 12d | Describe any sensitivity analyses | Not performed/not included |
| **Results** |  |  |
| 13a | Report numbers of individuals at each stage of study | Results – Sample sizes by cohort (Pages 9-10); Figure 2 caption (Page 18) |
| 13b | Give reasons for non-participation at each stage | Not explicitly discussed |
| 13c | Consider use of a flow diagram | Not included |
| 14a | Give characteristics of study participants | Supplemental File |
| 14b | Indicate number of participants with missing data | Supplemental File |
| 15 | Report numbers of outcome events or summary measures | Results – Tables and Figure 2 (Pages 10-11, Table captions on Page 19) |
| 16 | Provide unadjusted and, if applicable, adjusted estimates with precision | Results – Descriptive estimates with 95% CI shown in Tables (Pages 10-11) |
| 17 | Report other analyses (e.g., subgroup or sensitivity analyses) | Not included |
| **Discussion** |  |  |
| 18 | Summarize key results with reference to study objectives | Discussion – Opening paragraph (Page 11) |
| 19 | Discuss study limitations | Discussion – Limitations (Pages 14) |
| 20 | Provide overall interpretation of results considering objectives and limitations | Discussion – Summary and implications (Pages 11-14) |
| 21 | Discuss generalisability (external validity) | Discussion – Final paragraph (Page 14) |
| **Other Information** |  |  |
| 22 | Indicate funding sources and the role of funders | Acknowledgements / Funding (Page 18) |
| A1 | Ethical approval and informed consent described | Methods – Ethics section (Page 8) |
| A2 | Data availability statement | Data Statement (Page 18-19) |

Overview of statistical approaches applied to each primary and secondary outcome

**Supplemental Table S3**. Statistical approaches applied to primary outcomes.

| **Outcome** | **Approach** | **Numerator** | **Denominator** | **Analysis notes** |
| --- | --- | --- | --- | --- |
| **Full vaccination coverage** | Birth cohort | Children who were fully vaccinated by the age of 12 months | Children who survived to the age of 12 months by year of birth cohort | Analysis restricted to children who were observed in a Birhan HDSS round during the age of 11 to 13 months |
|  | Survey | Children who were fully vaccinated at any moment before being interviewed in and HDSS round, and before 24 months of age | Children aged 12-23 months on the 1^st^ of January of years 2020 to 2023 |  |
| **Coverage of specific vaccines** | Birth cohort | Children who were vaccinated with a specific vaccine by the age of 12 months regardless whether they survived to this age milestone | Children who survived to the age of vaccine eligibility by year of birth cohort | Analysis restricted to children who were observed in a Birhan HDSS round during the age of 11 to 13 months |
|  | Survey | Children who were vaccinated with a specific vaccine at any moment before being interviewed in and HDSS round, and before 24 months of age | Children aged 12-23 months on the 1^st^ of January of years 2020 to 2023 |  |
| **Timeliness of specific vaccines** | Birth cohort | Children who were vaccinated with a specific vaccine within four weeks of vaccine eligibility | Children who survived to the age of vaccine eligibility by year of birth cohort, and who were vaccinated for a specific vaccine | Analysis restricted to children who were observed in a Birhan HDSS round during the age of 11 to 13 months  Only data of children with vaccination card and vaccination dates were used |
|  | Survey | Children who were vaccinated with a specific vaccine within four weeks of vaccine eligibility | Children aged 12-23 months on the 1^st^ of January of years 2020 to 2023 who were vaccinated for a specific vaccine | Only data of children with vaccination card and vaccination dates were used |

***Notes:*** *Full vaccination - all vaccines required for full immunization <12 months in Ethiopia (see Table 1 of main manuscript), except for inactivated poliovirus vaccine (IPV) due to unavailability of data. Specific vaccines - full dose regimen (all recommended doses) of a specific vaccine (e.g. pentavalent vaccine) included in the full immunization package for children <12 months of age in Ethiopia.*

**Supplemental Table S4**. Statistical approaches applied to secondary outcomes.

| **Outcome** | **Approach** | **Numerator** | **Denominator** | **Analysis notes** |
| --- | --- | --- | --- | --- |
| **Proportion of zero-dose children** | Birth cohort | Children who were not vaccinated before being 12 months with at least one dose of pentavalent vaccine regardless of whether they survived to this age milestone | Children who survived to the age of vaccine eligibility for the first dose of pentavalent vaccine by year of birth cohort | Analysis restricted to children who were observed in a Birhan HDSS round during the age of 11 to 13 months |
|  | Survey | Children who did not receive the first dose of pentavalent vaccine before being interviewed and before the age of 24 months | Children aged 12-23 months on the 1^st^ of January of years 2020 to 2023 |  |
| **Proportion of drop-outs: pentavalent dose 1 to pentavalent dose 3** | Birth cohort | Children who were vaccinated with the third dose of pentavalent vaccine before being 12 months regardless of whether they survived to this age milestone | Children who received the first dose of pentavalent vaccine (following the birth cohort approach) | Analysis restricted to children who were observed in a Birhan HDSS round during the age of 11 to 13 months |
|  | Survey | Children who were vaccinated with the third dose of pentavalent vaccine at any moment before being interviewed in and HDSS round, and before 24 months of age | Children who received the first dose of pentavalent vaccine (following the survey approach) |  |
| **Proportion of drop-outs: pentavalent dose 1 to measles vaccine** | Birth cohort | Children who were vaccinated with measles vaccine before being 12 months regardless of whether they survived to this age milestone | Children who received the first dose of pentavalent vaccine (following the birth cohort approach) | Analysis restricted to children who were observed in a Birhan HDSS round during the age of 11 to 13 months |
|  | Survey | Children who were vaccinated with measles vaccine at any moment before being interviewed in and HDSS round, and before 24 months of age | Children who received the first dose of pentavalent vaccine (following the survey approach) |  |

Comparison of children with and without vaccination cards.

Children from the wealthiest families of the study area, living in Angolela woreda, in rural areas, and within <30 min of the nearest health facility showed higher rates of vaccination card availability. Certain health facilities (i.e. Tsigereda and Tere Health Centers) displayed increased rates of vaccination card availability compared to others (Table S4). Regarding kebele (not shown in the table), children living in Tsigereda followed by Seriti were those with the highest card availability rates (87% and 80%), while children from Chefanen and Sefi beret showed the lowest rates (32% and 37%, respectively).

**Supplemental Table S5.** Characteristics of children with and without vaccination cards (N=7,382).

| **Characteristics** | | **Card availability** | |
| --- | --- | --- | --- |
|  |  | **Yes** | **No** |
|  | | n (%) | n (%) |
| District (*woreda*) | Angolela | 1795 (64) | 1018 (36) |
|  | Kewet | 2562 (56) | 2007 (44) |
| Type of residence | Urban | 761 (52) | 691 (48) |
|  | Rural | 3596 (61) | 2334 (39) |
| Source of income | Farming | 3413 (59) | 2363 (41) |
|  | Other sources | 803 (58) | 582 (42) |
|  | Missing | 141 (64) | 80 (36) |
| Nearest health facility (HF) | Chacha Health Center | 982 (56) | 758 (44) |
|  | Tsigereda Health Center | 769 (77) | 227 (23) |
|  | Abaya atir Health Center | 1092 (53) | 953 (47) |
|  | Tere Health Center | 789 (67) | 396 (33) |
|  | Shewarobit Health Center | 195 (58) | 141 (42) |
|  | Shewarobit Hospital | 365 (45) | 446 (55) |
|  | Other | 30 (51) | 29 (49) |
|  | Missing | 135 (65) | 75 (35) |
| Walking time to nearest HF | <30min | 1044 (63) | 609 (37) |
|  | ≥30min | 3178 (58) | 2341 (42) |
|  | Missing | 135 (65) | 75 (35) |

These differences suggest that the results of the timeliness outcome may not be generalizable to the whole study sample. This may be underestimating the vaccination delays since children with cards are those from the wealthiest families and living closer to facilities.

Description of the sample used to estimate the outcomes

Supplemental Table S6 describes the total number of children born each year from 2018 to 2021, and the proportion of those who were used for the calculation of study outcomes using the birth cohort and survey approaches.

- Children who were observed in an HDSS round while being 11-13 months were included in the sample for the estimation of birth cohort outcomes.
- Children who were observed while being 12-23 months were included in the sample for the estimation of survey outcomes.

**Supplemental Table S6**. Distribution of live births identified in the Birhan Health and Demographic Surveillance System (HDSS) by birth cohort and data completeness status.

| **Birth year** | **Born children** | **Observed while being 11-13 months** | **Observed while being 12-23 months** |
| --- | --- | --- | --- |
|  | *N* | *n (%)* | *n (%)* |
| 2018 | 1897 | 619 (33) | 1694 (89) |
| 2019 | 1896 | 250 (13) | 1616 (85) |
| 2020 | 1719 | 478 (28) | 1567 (91) |
| 2021 | 1870 | 1148 (61) | 1648 (88) |

*Note: Column 2 represents all recorded live births; Columns 3 and 4 reflect subsets visited within specific age windows relevant for outcome measurement.*
